# Specific Features of the Functional Activity of Human Adipose Stromal Cells in the Structure of a Partial Skin-Equivalent

**DOI:** 10.3390/ijms25126290

**Published:** 2024-06-07

**Authors:** Diana Ya. Aleynik, Irina N. Charykova, Yulia P. Rubtsova, Daria D. Linkova, Ekaterina A. Farafontova, Marfa N. Egorikhina

**Affiliations:** Federal State Budgetary Educational Institution of Higher Education, Privolzhsky Research Medical University of the Ministry of Health of the Russian Federation, 603005 Nizhny Novgorod, Russia; daleynik@yandex.ru (D.Y.A.); irina-ch0709@yandex.ru (I.N.C.); rubincherry@yandex.ru (Y.P.R.); linckovadaria@yandex.ru (D.D.L.); ekaterina_farafontova@mail.ru (E.A.F.)

**Keywords:** adipose stem cells, mesenchymal stromal cell, scaffold, hydrogels, secretome, bio-materials, cell therapy, growth factors, scaffolds, conditioned media

## Abstract

Mesenchymal adipose stromal cells (ASCs) are considered the most promising and accessible material for translational medicine. ASCs can be used independently or within the structure of scaffold-based constructs, as these not only ensure mechanical support, but can also optimize conditions for cell activity, as specific features of the scaffold structure have an impact on the vital activity of the cells. This manuscript presents a study of the secretion and accumulation that occur in a conditioned medium during the cultivation of human ASCs within the structure of such a partial skin-equivalent that is in contact with it. It is demonstrated that the ASCs retain their functional activity during cultivation both within this partial skin-equivalent structure and, separately, on plastic substrates: they proliferate and secrete various proteins that can then accumulate in the conditioned media. Our comparative study of changes in the conditioned media during cultivation of ASCs on plastic and within the partial skin-equivalent structure reveals the different dynamics of the release and accumulation of such secretory factors in the media under a variety of conditions of cell functioning. It is also demonstrated that the optimal markers for assessment of the ASCs’ secretory functions in the studied partial skin-equivalent structure are the trophic factors VEGF-A, HGF, MCP, SDF-1α, IL-6 and IL-8. The results will help with the development of an algorithm for preclinical studies of this skin-equivalent in vitro and may be useful in studying various other complex constructs that include ASCs.

## 1. Introduction

It is difficult to imagine the development of current regenerative medicine without studying and applying mesenchymal stromal cells (MSCs). Due to their ability to undergo multilinear differentiation, their low immunogenicity, and their high proliferative activity, MSCs are considered the most promising cell material for cell therapy; for instance, for the activation of regenerative processes [1,2]. MSCs can be taken from many tissues: bone marrow, umbilical cord, dental pulp, and adipose tissue. [3,4]. Despite numerous studies on the specifics of obtaining MSCs, on their functional characteristics, and on the mechanisms of their action, their wide application means that research into such cell populations is still ongoing.

MSCs derived from adipose tissue (ASCs) have attracted special attention amongst the MSCs of various origins because such adipose cells are considered the most accessible and universally applicable tool for different types of cell therapy and tissue engineering [5,6]. ASCs meet the generally accepted criteria for MSCs [7,8], as they are characterized both by their high proliferative potential and their immunomodulative capacities [9,10]. Such ASC features, together with the possibility of obtaining large quantities of cellular material during regular liposuction procedures, have contributed to the fact that products and technologies based on the use of these cells are being actively developed and have already started to be applied in clinical practice [11,12,13,14,15]. The restoration of various skin defects is a promising area for the application of ASCs. Techniques based on the use of cultured skin cells (keratinocytes and fibroblasts) were initially used in cell therapy for these purposes [16,17,18,19,20,21]. Later, cultured skin cells began to be applied as part of the complex constructs that were being developed, which, in addition to the cellular component, included a carrier matrix [22,23,24]. The use of such constructs—so-called “skin-equivalents”—had promising clinical results [25,26]. However, their high susceptibility to infection, problematic application, high cost, and the complexity of the production technologies led to searches for other options for similar products; in particular, ones involving ASCs. It was established that the use of ASCs optimizes the process of skin regeneration through improved angiogenesis, and their synthesis of collagen and other components of the elastic matrix, such as oxytalan, elaunin, and elastin fibers [27,28]. In addition to the most important function, the enhancement of collagen synthesis, the ASCs secrete factors that are also known to improve some fibroblast characteristics, such as their proliferation and migration [29,30,31].

The effectiveness of ASCs in the restoration of skin defects has been repeatedly demonstrated in experiments on animal models. For instance, in full-thickness thermal burns, the stimulation of angiogenesis and epithelialization that followed localized ASC injections have been demonstrated in the mini-pig model [32]. The use of ASCs in an experimental sheep skin-burn wound model resulted in improved wound healing, increased blood flow, and enhanced VEGF synthesis [33]. ASC application also had a positive effect on skin quality during skin restoration as part of the scar forming process in mini pigs [34]. Comparing the use of the stromal vascular fraction of adipose tissue and ASCs in an animal model, Rapp et al. confirmed the predominant effectiveness of ASCs [35]. Lee H.C. et al. (2012) used cultured MSCs isolated from adipose tissue in the form of multiple intramuscular injections in 15 patients, both with ulcer formation and without, in cases of severe ischemia of the lower extremities, and demonstrated a positive effect and the absence of complications [36]. In a review, H. Jo et al. (2021) summarized data on the clinical use of MSCs and presented a number of studies showing the effectiveness and safety of using MSCs for the restoration of wound skin defects of various etiologies [37]. The majority of experts believe that the most promising therapy to optimize wound healing and for full-thickness skin restoration is based on the joint use of ASCs and scaffolds, which can not only render mechanical support for the cells, but also guarantees their functional activity. For these purposes, scaffolds based either on various natural biomaterials (collagen, elastin, fibrin) or on synthetic polymers have been proposed and studied in association with ASCs [38,39,40,41,42]. The use of such complex substitutes as skin equivalents in in vivo models demonstrated increased angiogenesis and wound re-epithelialization, with the deposition of newly grown collagen and thickened epidermal layers, resulting in improvements in the quality of the restored skin.

**Figure 1:**
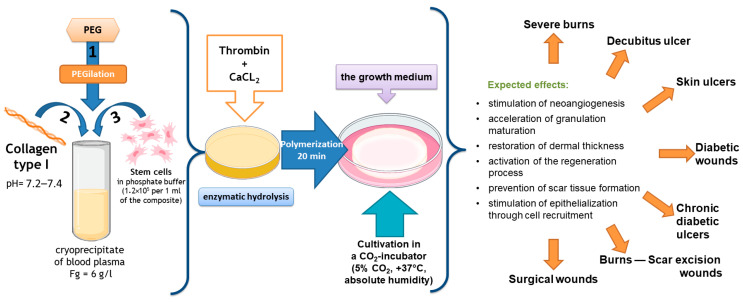
Scheme for the formation of the original partial skin-equivalent and possible areas of its application.

Our team of authors has developed a partial skin-equivalent made of a hydrogel scaffold based on natural biopolymers with encapsulated ASCs (Figure 1). During ASC cultivation in this scaffold structure, the cells retain their morphological traits and proliferative activity in vitro [43]. It is known that ASCs actively secrete many cytokines, growth factors, and extracellular matrix proteins involved in the wound-healing process, including vascular endothelial growth factor (VEGF), hepatocyte growth factor (HGF), insulin-producing growth factor (IGF), fibronectin, and interleukins (ILs)—IL-6, IL-8 [44,45,46,47,48,49].

Literature sources indicate that the secretory activity of ASCs can be influenced by various factors; for instance, the rigidity of the cell adhesion substrates and the topography of the surfaces of such substrates [50,51,52]. Biologically active scaffolds are of critical importance for ensuring optimal conditions for survival of the MSCs and can also influence their secretory characteristics by signal transmission from the outside, which is of particular importance during cell expansion and/or after cell delivery [53].

It is obvious that changing the conditions of ASC cultivation can affect specific features of the functioning of such cells within the structure of a given skin-equivalent.

Thus, alongside the characteristics of the carrier scaffold that forms this structure, these conditions are of paramount importance to the selection of the methods used in preclinical studies of the construct, starting from the initial stages in the in vitro system of research on experimental animal models, and, ultimately, for successful clinical application of the product.

Furthermore, in the case of complex constructs, it is desirable to choose methods that enable assessment and characterization of the cellular activity while maintaining cell viability without destroying the construct. We therefore reasoned that the simplest and most accessible approach would be to study the conditioned medium that formed during the cultivation of the construct and cells.

The purpose of the study was to study the specific features of secretion and accumulation of several proteins in the conditioned medium forming during human ASC cultivation in a partial skin-equivalent structure in order to select the optimal algorithm for assessment of the functional characteristics of the construct at the stage of preclinical studies in vitro.

## 2. Results and Discussion

**Figure 2:**
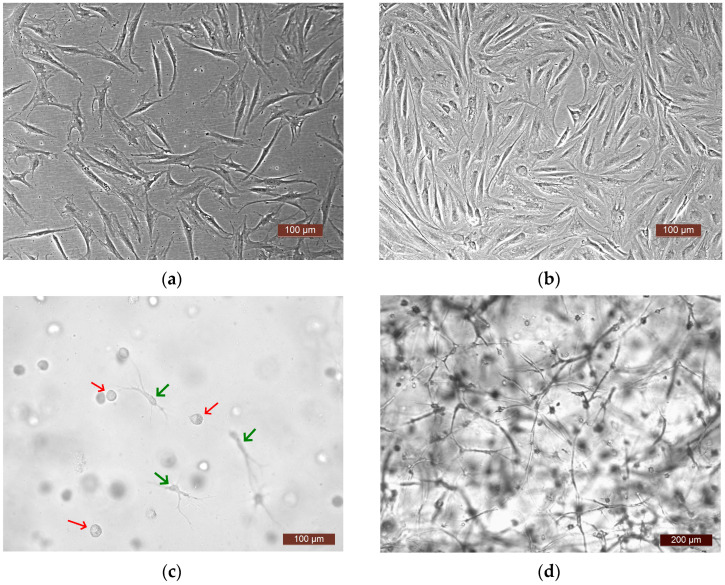
Representative photographs of ASCs. (**a**,**b**) ASCs on the plastic surface (plated cells—Ce series), phase contrast: (**a**) 24 h of cultivation, (**b**) 7 days of cultivation; (**c**,**d**) ASCs in the SE structure, light microscopy: (**c**) 24 h of cultivation, cells in the scaffold structure—spherical-shaped cells (red arrows), and cells that have formed processes (green arrows), (**d**) 7 days of cultivation (a cell network in the SE structure).

To analyze the functional activity of human ASCs, four experiments were carried out. In each one, a culture of ASCs from a different donor was used (donors No. 1, No. 2, No. 3, No. 4). The following series were generated for the study: Ce—ASCs growing directly on a plastic surface; SE—skin equivalent (ASCs within the structure of a three-dimensional hydrogel scaffold); and Sc—an acellular hydrogel scaffold. The methodology and conditions for all four experiments were similar (involving the same culture conditions, same batch of plastic, and same batch of media). The proliferative and secretory activity of the ASCs in the Ce and SE series was assessed over time. A comparative assessment of the dynamics of the release of various factors into the culture medium was also carried out in the Ce and SE series. As a control, the content of factors in the growth medium of the Sc series was assessed, which made it possible to evaluate the possible extraction of factors from the biologically active hydrogel scaffold. Furthermore, it should be noted that the microscopic approach used allowed observation of active cell proliferation in all experiments, both on the plastic surface—Ce series—and in the partial skin-equivalent structures—SE series. Visualization with phase-contrast microscopy and video archiving allowed the registration of changes in the states of the cells during cultivation both on the plastic (Figure 2a,b) and in the equivalent structure (Figure 2c,d). When cultivated on plastic, cells at all stages of cultivation had a typical spindle-like form, or, less often, a stellate form with pronounced processes. The cell nuclei were oval with clear boundaries and the cell cytoplasm was homogeneous. By Day 7 of cultivation, a subconfluent monolayer (up to 80%) was formed on the plastic. In the partial skin-equivalent structures (SEs), standard dynamic changes in the morphology of the ASCs were seen, as it changed from a spherical shape (Day 1—Figure 2c) to a straightened shape with pronounced processes and with the subsequent formation of a cell network (Day 7—Figure 2d).

**Figure 3:**
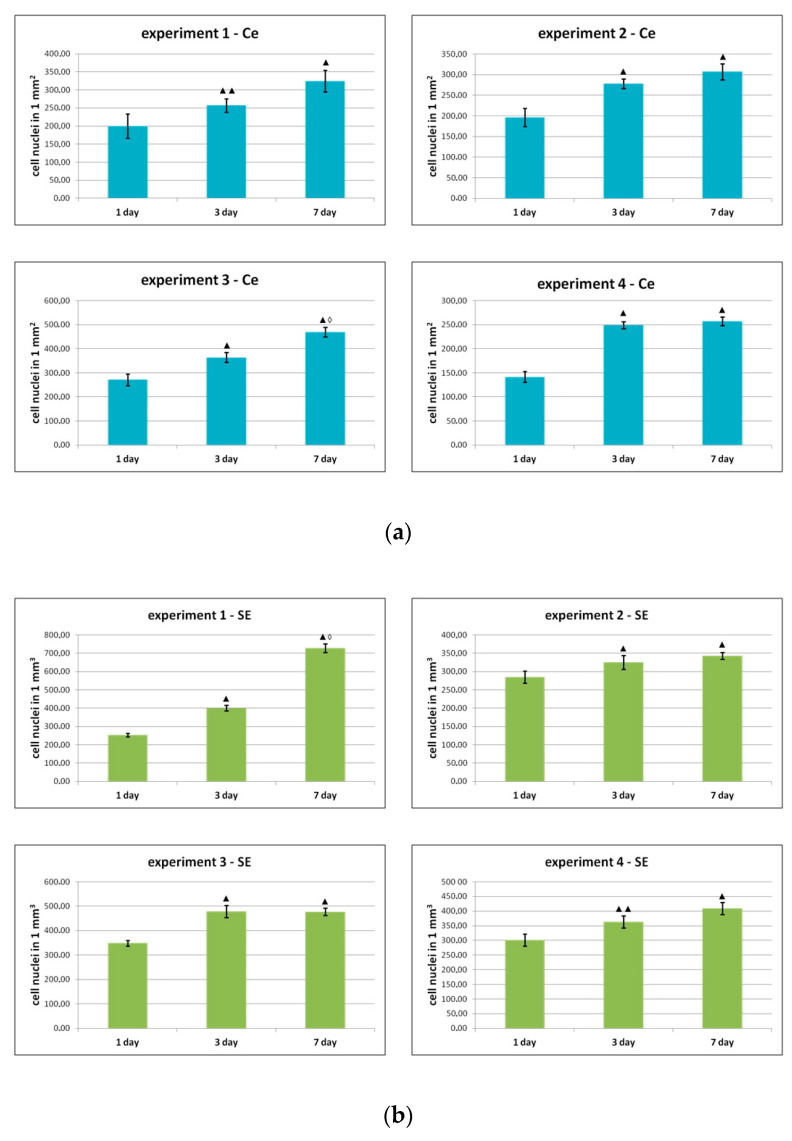
Change in the number of ASCs during cultivation (**a**) on plastic and (**b**) in the partial skin-equivalent structure. Note: ▲—*p* < 0.01 ▲▲—*p* < 0.05 compared with Day 1, ◊—*p* < 0.01 compared with Day 3 (Wilcoxon test); experiments 1–4—dynamics of growth of ASCs cultures from four different donors on plastic and within the structure of the skin equivalent.

During the process of observation, quantitative analysis of the cells, both in the Ce series and in the SE series, demonstrated pronounced proliferative activity of the ASC cultures (Figure 3) with a high proportion (95–98%) of retained, viable cells when cultivation occurred both on plastic and in the partial skin-equivalent structure.

As can be seen from the data presented in Figure 3, during the cultivation process, the number of cells increased statistically significantly after 1 and 3 days of cultivation in all experiments, both on plastic and in the structure of the skin equivalent. During subsequent cultivation, an increase in the number of cells was noted in all groups by Day 7 compared to Day 3, but only in two series (experiment 1, Se and experiment 3, Ce) were the differences between days 3 and 7 statistically significant.

One of the most important indicators of the functional activity of MSCs is their ability to secrete multiple proteins (interleukins, growth factors, etc.). It is obvious that the secretory function of MSCs is crucial for translational medicine. The results of research conducted over recent decades indicate that the clinical efficacy of MSCs is mainly associated with their paracrine activity [54,55,56,57,58].

**Figure 4:**
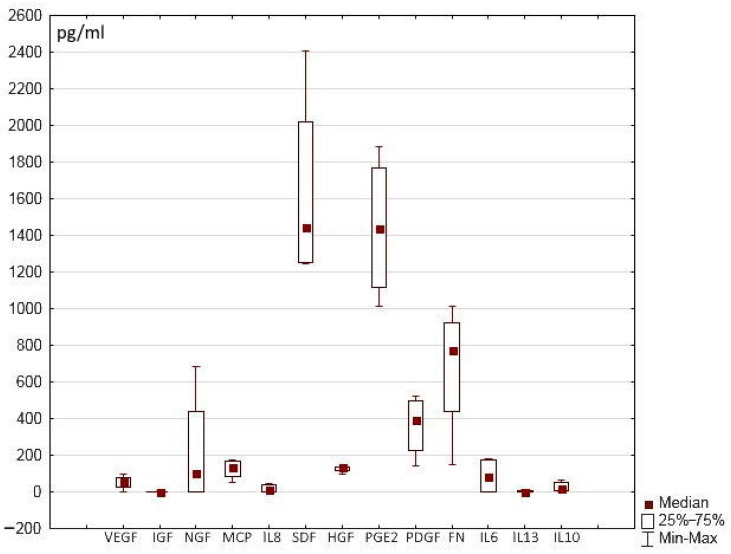
Content of trophic factors in cryoprecipitate samples.

The hydrogel scaffold presented in this work and its hydrogel scaffold-based partial skin-equivalents were formed using a blood plasma cryoprecipitate pool, the samples of which, in turn, contained a certain number of trophic factors. The data obtained showed that the content of these proteins varied in different samples of the pool (Figure 4). In the initial cryoprecipitate, some of these proteins could not be detected, or were present only in insignificant amounts (IL-10, IL-13, IL-6, IL-8, IGF, VEGF), whereas the content of other proteins was more significant (SDF, PGE-2, FN). Thus, it is necessary to consider whether these concentrations could have an impact on our findings in relation to our assessment of the secretory activity of the cells in the SE structures.

**Figure 5:**
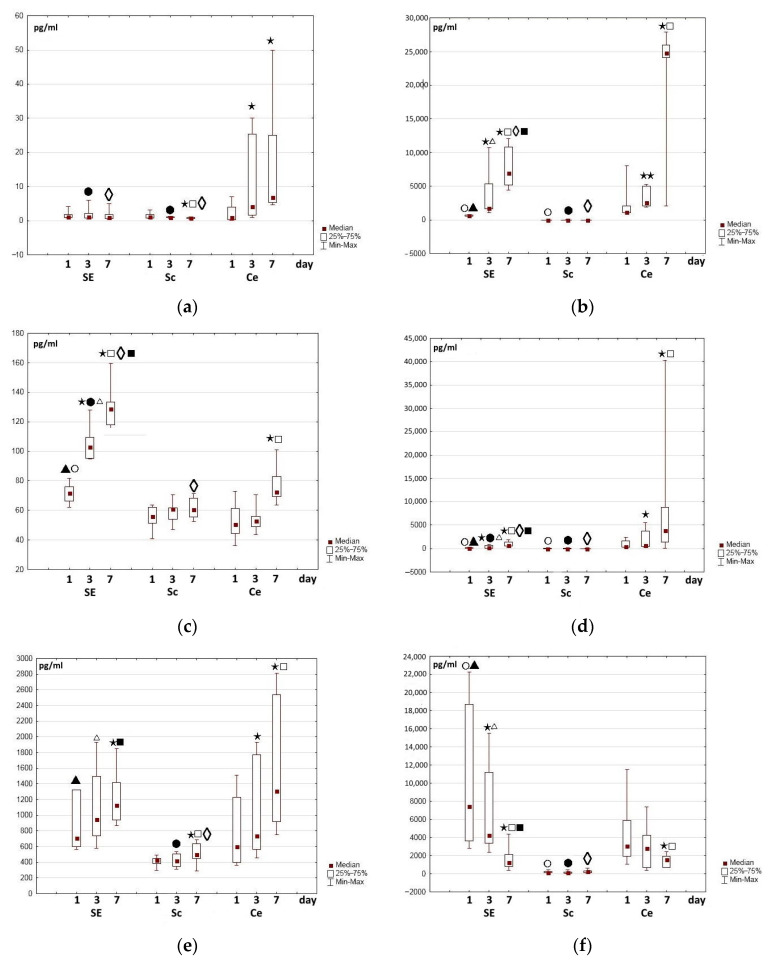
Changes in growth factor content in the medium during the cultivation of cells, partial skin-equivalents, and control scaffolds: (**a**) fibronectin, (**b**) VEGF-A, (**c**) HGF, (**d**) MCP, (**e**) SDF-1α, (**f**) PGE-2. Note: SE—partial skin-equivalents (scaffold with encapsulated cells—ASCs), Sc—cell-free scaffold, Ce—cells—ASCs cultured on plastic surface; ⋆⋆—*p* < 0.05; ⋆—*p* < 0.01 compared with Day 1, □—*p* < 0.01 compared with Day 3, Wilcoxon test; ○—*p* < 0.05—compared with Day 1, Ce; ●—*p* < 0.05 compared with Day 3, Ce; ◊—*p* < 0.05 compared with Day 7, Ce, ▲—*p* < 0.01 compared with Day 1, Sc, ∆—*p* < 0.01 compared with Day 3, Sc, ■—*p* < 0.01 compared with Day 7, Sc, Mann–Whitney test.

During each experiment, we assessed the content of all the studied growth factors and interleukins in the growth medium (MesenCult MSK basal medium and supplement; STEMCELL Technologies, Vancouver, BC, Canada). However, the levels of most of these proteins (FN, VEGF, MCP-1, HGF, IGF, NGF, IL-6, IL-13, IL-10) in the growth medium samples were undetectable. The content of others was insignificant: IL-8—5 [0; 6], SDF-1α—58 [50; 65.7], PGE-2—38 [0; 41] pkg/mL (the results are expressed as the Me [Q1; Q3] where Me is the median of the analyzed parameter and [Q1; Q3] are the 25th and 75th quartile values, respectively). At the same time, in the samples of the experimental series (SE, Ce), the content of certain proteins (FN, VEGF, MCP-1, HGF, IL-6, IL-8, SDF-1α, PGE-2) ranged from hundreds to thousands of pg/mL (Figure 5). When assessing the experimental results, we therefore neglected the content of these proteins in the initial growth medium.

Determination of the trophic factor content of the conditioned medium during ASC cultivation on plastic and in the partial skin-equivalent structures showed that their accumulation dynamics were also different. Not all of the proteins were detected in the media of every series of experiments (SE, Sc, Ce) at all control points of the study; particularly the nerve growth factors (NGFs), IL-10, and IL-13.

Twenty-four hours after the start of the study, insulin-producing growth factor (IGF), a protein involved in angiogenesis and regeneration processes, could not be detected in the entire series of experiments. Likewise, no IGF was detected during observations of the medium of cell cultures on plastic. However, in the control series of acellular scaffolds (Sc series) on Day 3, its level ranged from 1.13 to 4.06 pg/mL of IGF, while in the SE “partial skin-equivalent medium” series, the level was 0.43–2.52 pg/mL. By Day 7 of the study, the IGF content had slightly increased and reached 5.44–7.41 pg/mL in the Sc control series medium and 2.65–5.15 pg/mL in the SE medium. Here, the IGF content was always slightly higher in the medium of the control Sc series than in the medium of the partial skin-equivalent series.

Similar results were obtained for the determination of platelet-derived growth factor (PDGF), which, 24 h after the beginning of the study, was detected only in the medium of the Sc control series. Later, its content in the medium of the control series varied from 60 to 120 pg/mL in different experiments. During cultivation, by Day 7, an insignificant amount (8–10 pg/mL) of this protein was also noted in the SE series. It can be assumed that the IGF and PDGF present in the medium of the Sc and SE samples are associated with their extraction from the scaffolds.

Considering the low content of the above-mentioned proteins in both the samples of the initial cryoprecipitate and in the conditioned medium of the experimental series, it can be assumed that determination of these factors is not feasible as a marker for the functional activity of the partial skin-equivalent samples.

One of the main structure-forming multifunctional proteins of the extracellular matrix, fibronectin, was chosen for study from the overall list of such matrix proteins; it has an important role in the process of dynamic remodeling, especially in wound healing [59,60]. Fibronectin is involved in the stimulation of both cell proliferation and migration [61,62]. Wang et al. (2019) showed that preliminary covering of the wound bed with fibronectin as part of the treatment of full-thickness wounds stimulates their healing. The authors found that fibronectin ensured faster collagen formation during the early stages of wound treatment, as well as regulation of the gradual degradation of collagen as the wound healed. As a result, this contributed to improving the quality of the restored skin [63]. In our study, a sufficiently high level of fibronectin was recorded in all experiments in the Ce series (cells on the surface of the plastic) and in the SE series even after 24 h of observation. Furthermore, in each experiment, the content of fibronectin in the “cell medium” was higher than in the “partial skin-equivalent medium”. During cultivation in the Ce series, the content of fibronectin gradually increased in all experiments. Meanwhile, in the SE series, the content of fibronectin varied, although it remained approximately at a level that was comparable with the changes in this protein in the medium of the control series (Figure 5a). A certain proportion of the content of fibronectin in the medium of the Sc and SE series was probably due to its relatively high level in the cryoprecipitate used. This assumption was confirmed by an observed increase in the fibronectin content in the medium of the control scaffolds and the corresponding partial skin-equivalents with any increased level in the cryoprecipitate that was used for their formation. One can assume that, during cultivation, a certain amount of fibronectin escaped from the scaffold structure, as well as from the partial skin-equivalent, into the conditioned medium.

The relatively constant recorded fibronectin levels in the partial skin-equivalent medium (SE) during cultivation, together with the confirmed active proliferation of the ASCs within its structure were most likely due to the concurrent consumption of fibronectin synthesized by the ASCs, as it was used in the processes occurring in the partial skin-equivalent structures. For instance, densification of the partial skin-equivalent structures was observed during the cultivation of the ASCs, and had also been demonstrated using transmission electron microscopy in the authors’ previous work [64]. The current authors believe that this process is a result of the deposition of collagen matrix, as described in the literature, being facilitated by the pericellular structure formed by the fibronectin [61,62]. It is known that the fibrillar structure of fibronectin, which acts as a biological adhesive and mediates interactions between cells and other proteins, ensures enhanced perimetric formation [61,62]. Thus, the results indicate that, despite the high functional activity of the ASCs within the structure of the studied partial skin-equivalents, the content of fibronectin in the conditioned medium cannot be used to assess the functional activity of such a construct.

In addition to the levels of fibronectin in the media, the authors assessed changes in the concentrations of a number of trophic factors synthesized by ASCs that, as well as being involved in the regulation of proliferation and migration processes, also have pro-angiogenic and antiapoptotic impact. These were the following multifunctional proteins: VEGF-A, HGF, MCP, SDF-1α, IL-6 and IL-8. Differences in the levels and dynamics of accumulation of these proteins in the conditioned medium during cultivation were identified, both in the media of the experimental series and in the media of the control series.

Vasculo-endothelial factor (VEGF) is considered to be the most important and key pro-angiogenic factor and has been studied for decades [65,66]. In mammals, the VEGF family includes five factors (VEGF-A, -B, -C, and -D and placental growth factor), among which VEGF-A is the main one. VEGF-A acts as an effective pro-angiogenic mediator, and participates in the proliferation, migration, and regeneration processes that regulate wound healing [67,68]. During the entire study period, in the control Sc series, VEGF-A was almost undetectable or its level was minimal, as was its presence in the initial cryoprecipitate. However, a significant amount of VEGF-A was detected in the experimental series media even as soon as one day after cell seeding and the formation of partial skin-equivalents (Figure 5). Here, in the experimental Ce series in each individual experiment, the level of VEGF-A was always higher than in the SE series. During cultivation, the content of VEGF-A increased in the experimental series in all experiments, but it was greater in the Ce series (Figure 5b).

The data obtained in this series of experiments using four different cultures of ASCs are similar to the results of earlier studies of other cultures of human ASCs [69]. One can assume that the greater accumulation of VEGF-A in the conditioned media of the Ce series, compared to the SE series, over the 7 days of the study is related to the observed later activation and increase in cell secretion in the partial skin-equivalent structures. Moreover, it is obvious that the secretory activity of the ASCs may depend on the characteristics of the scaffold. For example, L. Cai et al. (2016) demonstrated the dependence of the pro-angiogenic (VEGF, PLGF synthesis) activity of MSCs on the density of the substrate on which, or within the structure of which, they functioned in vitro [70].

Unlike VEGF-A, 24 h after the beginning of the study, hepatocyte growth factor (HGF) was registered in insignificant amounts in the media of all series (Figure 5c), although prevailing in the SE series. With further cultivation in the SE series, a significant increase in the content of this protein in the medium was noted both on Day 3 and Day 7 of the study. Here, in the Ce series, the HGF content increased less markedly and was expressed only by Day 7 of cultivation (Figure 5c). In the control Sc series, the HGF level remained practically unchanged or fluctuated only very slightly during cultivation.

Monocytic chemoattractant protein (MCP) is another trophic factor involved in angiogenesis, and it has an anti-apoptotic effect [71,72,73]. Determination of its content in the growth media of the control scaffolds, the Sc series, indicated that while it was insignificant in all experiments, it varied slightly during incubation, and was comparable to the content of MCP in the cryoprecipitate. In all experimental series, the content of MCP 24 h after the beginning of cultivation was higher than in the medium of the control, Sc, series. The MCP level in the SE series turned out to be lower than in the Ce series. During the cultivation of both the partial skin-equivalents and of the cells on plastic, the content of MCP in the growth medium increased; during the cultivation of the cells on plastic, this increase was greater than during the partial skin-equivalent cultivation (Figure 5d). The direction of the registered changes was similar to those recorded for VEGF-A.

Determination of the stromal cell factor (SDF-1α), indicated that its level 24 h after seeding of the cells on the plastic in the conditioned cell medium was approximately equal to or slightly lower than that in the partial skin-equivalent medium. Meanwhile, in the control, Sc, series, the SDF-1α level was lower, although slight variations were seen in its dynamics during cultivation. In the experimental SE and Ce series, a clear (up to 50%) increase in protein content was noted by Day 7 of cultivation and the increase was even greater in the Ce series (Figure 5e).

The study of SDF-1α secretion by skin repair equivalents is of particular importance, as it is known that SDF-1α is one of the critical proteins that determine cellular events in human skin. SDF-1α is both a constitutive, and the most expressed, protein involved in the migration of human skin cells in both normal and damaged tissues [74,75,76]. A number of studies have demonstrated that ASCs overexpress SDF-1α to recruit cells to the damaged skin area from the surrounding tissues [76,77]. In this respect, SDF-1α stimulates the paracrine, proliferative, and migratory capacities of the recruited cells [78,79,80,81]. 

The data obtained demonstrate the active secretion of SDF-1α by ASCs in the structure of the partial skin-equivalents under study, providing evidence of its prospective use in the restoration of skin defects. Moreover, the authors registered a clear dynamic of SDF-1α accumulation in the conditioned media during the cultivation of ASCs on plastic. Considering these results, one can state the likely value of inclusion of this protein in cell secretion assessments in order to characterize the functional activity of cell cultures and partial skin-equivalents to be used for the restoration of skin lesions of various origins.

The roles of the interleukins IL-6 and IL-8 in the cascades of inflammation and regeneration processes are well known. For example, in studies on ischemia models [82] and on skin wound healing [83] by depleting IL-6 and IL-8 from the ASC’s secretome, a significant decrease in regenerative activity was demonstrated. Moreover, the angiogenic role of IL-8 has been shown for wound healing [84]. IL-6, in turn, has been shown to be involved in the regulation of angiogenesis [85] in addition to its capacity to inhibit apoptosis [86]. Taking into account the known activity of these interleukins in a variety of cellular processes, their study was therefore included in this work.

It turned out that changes in IL-6 and IL-8 were generally similar. In samples of the cryoprecipitate that was used to form the scaffolds and partial skin-equivalents, the content of IL-6 and IL-8 was minimal, as was their content in the medium of the Sc control series. In the medium of all experimental series, significant amounts of IL-6 and IL-8 were seen even after just 24 h, and their levels increased greatly during further cultivation of the samples. The content of IL-6 in the medium of the experimental series increased, starting from the first stage of the study (3000–6000 pg/mL at 24 h), and reached 14,000–30,000 pg/mL by Day 7. No statistically significant differences were registered between the levels for the Ce and SE series. Unlike IL-6, after 24 h the content of IL-8 in the SE series was higher than in the Ce series, amounting to 3947–6134.67 pg/mL; during further cultivation, it increased in each experiment up to 10,000–30,000 pg/mL. In the Ce series, these dynamics of IL-6 during cultivation were expressed in all experiments, but not that significantly: from 1412 to 6119 pg/mL after 24 h to 5140–7797 pg/mL on Day 7 of the study.

The dynamics of changes in the content of prostaglandin E (PGE-2) are of interest. In the control Sc series, PGE-2 was recorded at all stages of the study. Its content varied, but did not exceed 350 pg/mL (Figure 5f). These values differed significantly from the levels of PGE-2 in the media of the respective experimental series. The level of PGE-2 in the control series medium was, most likely, due to gradual escape of the protein from the scaffold samples and to its high original level in the initial cryoprecipitate. After 24 h, a significant content of PGE-2 was identified in the medium of all experimental series (Ce, SE) in all experiments. During cultivation, the content of PGE-2 in the conditioned medium of the experimental series decreased in all experiments, regardless of the initial level, and had fallen even more markedly by Day 7 (Figure 5f).

To understand the dynamics of the changes in the PGE-2 level in the medium of the experimental samples, one should consider its functions and role in various processes. PGE-2 is a mediator in many physiological and pathological functions. It can be produced by various cells in the body, and MSCs are among the most important producers of this lipid [87,88,89]. PGE-2 is known as an effective immunomodulator. Being produced by MSCs, PGE-2 not only interacts with the target cells (dendritic, T-cells, NK-cells), but also has an autocrine impact on MSC proliferation by activating the EP2 receptor. [90]. It is already known that the COX-2/PGE-2 axis plays an important role in maintaining the basic vital functions of MSCs, such as their capacity to proliferate, migrate, and differentiate. Recent studies have shown that PGE-2 synthesized by MSCs has a positive effect on the formation of unique niches that, for example, enhance hematopoietic processes [91]. It is evident that the importance of PGE-2 for skin damage repair processes is highly significant. For instance, in experiments on animal models, an acceleration of the healing of skin wounds has been noted with an increase in the level of PGE-2 [92,93]. PGE-2 is further involved in restoration processes following a variety of pathologies, through its anti-inflammatory function, its facilitation of angiogenesis, and particularly in the prevention of scarring, which makes it a promising agent for the treatment of excised skin wounds [94]. PGE-2 can enter the extracellular medium both by passive diffusion from the producer cells and by being actively transported by the prostaglandin transporter MRP4 (multidrug resistance protein 4) [95].

The high level of PGE-2 in the medium of the experimental series as seen in this study 24 h after subculturing or after the formation of partial skin-equivalents was probably the result of active release of the protein from the cells. The simultaneous progress of cell proliferation and intercellular interaction could be observed both during ASC cultivation within the scaffold structure and during their cultivation on plastic. One can assume that such intensification of cellular processes in the ASCs probably resulted from a greater amount of PGE-2 acting via the autocrine path, and that this came from the conditioned medium. These results deserve close attention and further detailed study. However, the identified dynamics of the PGE-2 content, which demonstrated an abrupt decrease during cultivation in conditioned media of both the cells on the surface of the plastic (Ce) and those in the partial skin-equivalents, make its determination inappropriate for use in assessment of the functional activity of ASCs.

Hence, the results of this study indicate that the presence of certain factors (FN, SDF-1α, PGE-2) in the medium surrounding the control, acellular, scaffolds is most likely due to their significant presence in the original cryoprecipitate that was used in the formation of the scaffolds and partial skin-equivalents. The accumulation of some trophic factors stimulating angiogenesis and participating in the processes of proliferation and regeneration was seen in the conditioned medium of both the partial skin-equivalents and the cells on the surface of the plastic (Ce) during cultivation. Furthermore, it was noted that higher concentrations of some trophic proteins (VEGF-A, MCP-1, and SDF-1α) prevailed in the conditioned “cell media”, whereas others (HGF, IL-8) dominated in the conditioned “partial skin-equivalent media”. The identified differences can definitely be related to the characteristics of the synthesis of various proteins under different conditions, as well as to the characteristics of the proteins themselves. Explanations of these differences require further research.

## 3. Materials and Methods

### 3.1. ASC Culture

ASCs were isolated from the adipose tissue of four healthy donors (25–30 years old) during scheduled cosmetic liposuction procedures. The patients had no oncological or autoimmune diseases, nor tuberculosis, or transmissible infections in their anamneses. The donors were also examined for transmissible infections, and each donor of biomaterial provided voluntary informed consent to participate in the study. The sampling of biomaterial, collection of cell cultures, and their subsequent use for in vitro studies were conducted in accordance with the guidelines of the Declaration of Helsinki and approved by the local ethics committee of the Federal State Budgetary Educational Institution of Higher Education, the “Privolzhsky Research Medical University” of the Ministry of Health of the Russian Federation (FSBEI HE PRMU MOH Russia) (Nizhny Novgorod, Russia; 10 March 2021, protocol No. 5 and 30 June 2023, protocol No. 9).

The 3–4 passage ASC cultures for the study were received and characterized in the biotechnical laboratory of the University Clinic of the FSBEI HE PRMU of the Ministry of Health of the Russian Federation. All cultures were sterile and not contaminated with mycoplasmas or viruses. Before the experiment and at all its different stages, the cells of the cultures were morphologically homogeneous and were represented by fibroblast-like cells with clear contours, pronounced processes, and dense nuclei. The cells formed subconfluent monolayers (70–80%) with a characteristic curl-like pattern. The cell viability in the cultures was 97–98%.

Determination of the immunophenotype of the ASCs was carried out by multicolor analysis using a direct immunofluorescence reaction. We used a panel of monoclonal antibodies: CD 90FITC, CD 105PE, CD 73 PE, CD 45PC5, CD 14PC5, CD 10PC7, CD 34PC7 and CD HLA-DR PC7 (Bacman Coulter, Brea, CA, USA) with appropriate isotype controls on a FACS CANTO cytofluorimeter II (Becton Dickinson, Franklin Lakes, NJ, USA). The measurement setup was performed once and standardized using a BD™CS&T Beads (BD Cytometer Setup and Tracking Beads). The stained cells were incubated for 30 min and washed for further immunophenotype determination. The results were expressed as the proportion of cells carrying the corresponding marker (percentage). The cell phenotype of all cultures was typical for MSCs: 98% of the cells expressed CD90 = 98%, CD105 = 100%, CD73 = 100% and CD10 = 100% while not expressing CD45− = 0%, CD14− = 0%, CD HLA-DR− = 0% or CD34− = 0%.

**Figure 6:**
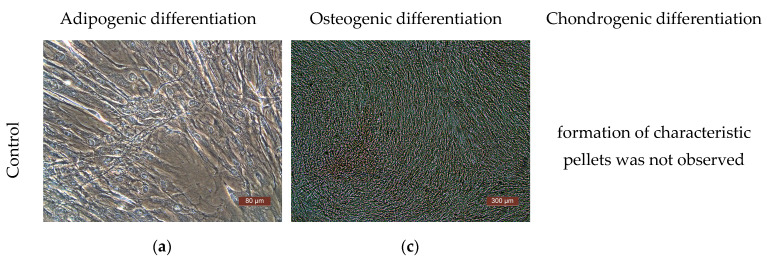

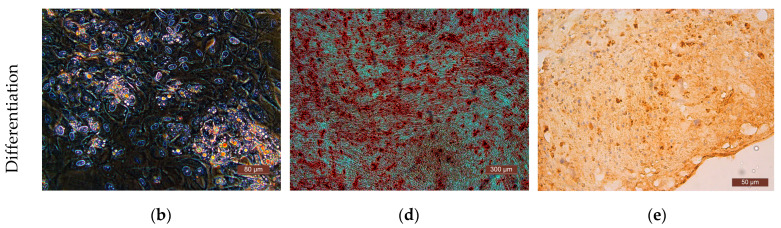
Representative photographs of human ASCs at various stages of differentiation (light microscopy). (**a**) Control—culture of ASCs without differentiation medium; formation of fat vacuoles is not observed. (**b**) Experiment—culture of ASCs after cultivation in a differentiation medium; fat vacuoles stained with Oil Red are clearly visible in culture cells, which demonstrates the ability of ASCs for undergoing adipogenic differentiation. (**c**) Culture of the control series of ASCs is presented in the form of a confluent monolayer formed by morphologically homogeneous spindle-shaped cells; there are no calcium deposits. (**d**) Culture of ASCs of the experimental series after cultivation in differentiation medium; the culture is a confluent monolayer; calcium deposits stained with alizarin red are clearly visualized. (**e**) Staining of type II collagen deposits with polyclonal antibodies (Abcam, ab34712) in the spheroid formed by cells of the ASC culture cells after cultivation in a chondrogenic differentiation medium; the formation of small spheroids and the deposition of type II collagen cells indicate chondrogenic differentiation of the ASCs.

The differentiation potential of the cells was assessed on their 3rd passage cultures. To obtain evidence of differentiation, the Human Mesenchymal Stem Cell Functional Identification Kit (R and D systems, Minneapolis, MN, USA) was used. The following specific dyes were used to stain lipid vacuoles—Oil Red (Sigma, USA); to detect calcium salts in the process of differentiation into osteoblasts—alizarin red (Sigma, USA); to detect type II collagen in spheroids—polyclonal antibodies (Abcam, ab34712). Cells of all the cultures used in the study demonstrated their ability to differentiate into adipocytes, chondrocytes, or osteocytes (Figure 6).

### 3.2. Formation of Scaffolds and Partial Skin-Equivalents

The acellular scaffolds (Sc series) and partial skin-equivalents (SE series) for the study were formed on the basis of a cryoprecipitate of blood plasma and fish collagen under conditions of enzymatic hydrolysis, in line with our previously described technique [43]. In order to determine their content of cytokines and growth factors, samples were taken from all the cryoprecipitate patterns that were used in the formation of the Sc and SE series.

### 3.3. Microscopy

The dynamics of the growth of the cells on plastic and the state of the cells in the partial skin-equivalent sample structures were systematically monitored using light microscopy and phase-contrast microscopy (Leica DMI 3000 B inverted microscope, Leica Microsystems, Wetzlar, Germany, integrated with LAS v. 4.3 software, Leica Microsystems, Wetzlar, Germany), and saved in a video archive for further analysis.

### 3.4. Assessment of Functional Activity of the ASCs

All studies were performed in quadruplicate using four ASC cultures obtained from different donors. Thirteen SEs were formed for each experimental series in order to assess the functional activity of the ASCs within the SE structure. Ten Sc cultures were simultaneously set up for each control study. Each sample (SE series and Sc series) was placed in a Petri dish (20 cm^2^, Corning). At the same time, ASCs from the same culture, with a density of 10 thousand cells/cm^2^, were seeded onto 10 Petri dishes (20 cm^2^, Corning) on the surface of the plastic (Ce series). A special serum-free MesenCult MSK basal medium and supplement (STEMCELL Technologies, Vancouver, BC, Canada) with added penicillin (33.3 µg/mL) and streptomycin (55.5 µg/mL) (LLC PanEco, Moscow, Russia)) antibiotics was used as the growth medium, and then the SE series, Sc series, and ASCs on the surface of the plastic (Ce series) were cultivated under standard conditions in a CO_2_ incubator (5% CO_2_, 37 °C, absolute humidity). The initial volume of the growth medium was 6 mL per dish.

During the study, the 10 samples of SE, Sc and ASC cultures were cultivated on dishes for up to 7 days in the CO_2_ incubator in a wetted medium at 5% CO_2_ and a temperature of 37 °C without changing the medium.

At control points (Days 1, 3, and 7 after formation of the partial skin-equivalents and the seeding of the cells), samples of the growth medium were taken during cultivation, aliquoted in 500 μL quantities into Eppendorf tubes and frozen at −40 °C for subsequent determination of their trophic factor content. The study of each aliquot was carried out in 2–3 replicates.

### 3.5. Enzyme Immunoassay

The protein levels in the growth medium were determined by means of an enzyme-linked immunoelectrodiffusion assay using the following ELISA kits: ThermoFisher Scentific (Bender MedSystems, Wien, Austria)—IL-6, IL-10, PDGF-BB, HGF, VEGF-A, MCP-1, IL-13 and IL-8; Mediagnost (Reutlingen, Germany)—IGF-1; Technoclone (Wien, Austria)—Fibronectin (FN); RayBiotech (Peachtree Corners, GA, USA)—β-NGF-1; R&D Systems (Minneapolis, MN, USA)—Prostoglandin E 2 (PGE-2), CXCL12/SDF-1α. The samples were analyzed in accordance with the manufacturers’ recommendations. The measurements were conducted using an INFINITI F50 photometer (Tecan Austria, Grödig, Austria) with Magellan software V7.2 (Tecan Austria, Grödig, Austria), which enables automatic building of a calibration curve and determination of the concentration of the substances. Moreover, for each experiment, the content of proteins in the initial cryoprecipitate and in the used growth medium were determined.

### 3.6. Quantitative Analysis of Cells including Assessment of Their Viability

The analysis was conducted using a minimum of three SEs in parallel with the assessment of secretory activity at the following control points: 24 h after SE formation (Day 1), on Day 3 and on Day 7, by taking samples of the cells in the SE structures for quantitative analysis. Cells in the partial skin-equivalent structures were counted using an original technique [43]. This technique is based on the application of intravital staining of the nuclei with Hoechst 3334 fluorochrome, followed by wide-field fluorescence microscopy over 10 fields of view using the Z-stack function, counting of the cell nuclei on the cross-linked Z-stack microphotographs, and recalculation of the number of cells per 1 mm^3^. For this purpose, parts of the analyzed SE samples were stained with fluorochromes on the control days and examined. NucGreen™ Dead488 Invitrogen™ fluorescent dye (477 nm excitation wavelength, 525 nm emission wavelength) was used to determine the number of dead cells. At the specified times, the cells in three Petri dishes with the ASC control cultures (plated cells) were stained with the same fluorescent dyes (the Hoechst 3334 fluorochrome and NucGreen™ Dead488 Invitrogen™), and at least 10 microphotographs were taken for each dish; then, during cultivation on a plane, the cell density (pcs/mm^2^) and the proportion of dead cells were determined.

To implement the quantitative analysis methods, the wide-field fluorescence microscopy was performed using a Cytation 5 imager with Gen 5 Image+ software 3.8 (BioTek, Winooski, VT, USA).

### 3.7. Statistical Analysis

Statistical analysis was conducted by means of the STATISTICA 6.0 software system (TIBCO Software Inc., Palo Alto, CA, USA). Nonparametric statistics methods, including the Mann–Whitney test and the Wilcoxon test for paired comparisons were applied.

## 4. Conclusions

During cultivation within the structure of the partial skin-equivalents under study, as well as during their cultivation on plastic, ASCs retain their functional capacities: they actively proliferate and secrete various proteins that can accumulate in the conditioned media. Our comparative study of the conditioned media during cultivation of ASCs on plastic and within the structure of the partial skin-equivalents showed that the release and accumulation of secretory factors in the medium are subject to different dynamics under different conditions of cell functioning.

The data obtained demonstrated that, considering the low content of each of the nerve growth factors (NGFs) IL-10, IL-13, IGF, and PDGF both in the samples of the initial cryoprecipitate and in the medium of the experimental series, it is not feasible to use determination of the levels of these factors as markers for assessing the functional activity of the studied partial skin-equivalents. The high, though fairly consistent level of fibronectin one of the main proteins of the extracellular matrix in the conditioned medium of the partial skin-equivalents, which also prevents us using it to assess the latter’s functional activity, because fibronectin, synthesized by the ASCs in the partial skin-equivalent structures, is actively used to remodel the structure, and therefore it does not accumulate further in the conditioned medium. The PGE-2 content in the conditioned media during the cultivation of ASCs on plastic and within the partial skin-equivalent structures decreases dynamically; therefore, it cannot be used to assess the functional activity of the cells.

It is most feasible to use the trophic factors—VEGF-A, HGF, MCP, SDF-1α, IL-6, and IL-8—as optimal markers for assessment of the ASCs’ secretory function in the structure of the original partial skin-equivalents; these factors demonstrated visible dynamics of accumulation in the conditioned media during cultivation of both the partial skin-equivalents and the cells on plastic.

Thus, proteins synthesized by ASCs under different conditions can both accumulate in the medium and be consumed from it. This is probably related not only to the cultivation conditions but also to the characteristics of the factors themselves, as well as being influenced by the content and structure of the partial skin-equivalents. The structure of the skin-equivalents can also determine the extent of the escape of certain factors. Moreover, one cannot exclude their potential impacts on the processes of synthesis nor the effects resulting from individual characteristics of the ASC culture used; hence, the level of any particular protein in experiments with different cultures of ASCs may differ slightly.

Our experience with the skin-equivalent should be of use to specialists developing tissue-engineered constructs and methods for assessing their quality at various stages of in vitro studies.

## Data Availability

The data presented in this study are available in the article.
